# Thermal- and Oxidative Stress Causes Enhanced Release of NKG2D Ligand-Bearing Immunosuppressive Exosomes in Leukemia/Lymphoma T and B Cells

**DOI:** 10.1371/journal.pone.0016899

**Published:** 2011-02-25

**Authors:** Malin Hedlund, Olga Nagaeva, Dominic Kargl, Vladimir Baranov, Lucia Mincheva-Nilsson

**Affiliations:** Division of Clinical Immunology, Department of Clinical Microbiology, Umeå University, Umeå, Sweden; Centre de Recherche Public de la Santé (CRP-Santé), Luxembourg

## Abstract

Immune evasion from NK surveillance related to inadequate NK-cell function has been suggested as an explanation of the high incidence of relapse and fatal outcome of many blood malignancies. In this report we have used Jurkat and Raji cell lines as a model for studies of the NKG2D receptor-ligand system in T-and B cell leukemia/lymphoma. Using real-time quantitative RT-PCR and immunoflow cytometry we show that Jurkat and Raji cells constitutively express mRNA and protein for the stress-inducible NKG2D ligands MICA/B and ULBP1 and 2, and up-regulate the expression in a cell-line specific and stress-specific manner. Furthermore, we revealed by electron microscopy, immunoflow cytometry and western blot that these ligands were expressed and secreted on exosomes, nanometer-sized microvesicles of endosomal origin. Acting as a decoy, the NKG2D ligand-bearing exosomes downregulate the *in vitro* NKG2D receptor-mediated cytotoxicity and thus impair NK-cell function. Interestingly, thermal and oxidative stress enhanced the exosome secretion generating more soluble NKG2D ligands that aggravated the impairment of the cytotoxic response. Taken together, our results might partly explain the clinically observed NK-cell dysfunction in patients suffering from leukemia/lymphoma. The adverse effect of thermal and oxidative stress, enhancing the release of immunosuppressive exosomes, should be considered when cytostatic and hyperthermal anti-cancer therapies are designed.

## Introduction

Several immune mechanisms participate in protecting the host against cancer. In these mechanisms the NKG2D receptor-ligand system plays a key role. The activating NK cell receptor Natural Killer Group 2, member D (NKG2D) and its human ligands, the MIC (MHC class I Chain-related proteins A and B) and ULBP (UL-16 Binding Proteins) 1–6, also known as RAET1, comprise a powerful cytotoxic system by which foreign, transformed or infected cells are eliminated from the body [1]. In murine studies, NKG2D receptor-dependent elimination of tumor cells expressing NKG2D ligands has been well-documented both *in vitro* and *in vivo* [1]–[6]. In humans, a specific NKG2D gene polymorphism has been associated with susceptibility to cancer [7]. So far, little is known about the regulation and expression of human NKG2D ligands (NKG2DL) in normal and transformed cells, except that they share the common property of induction by a variety of stresses [8]. In cancer patients, NKG2DL are constitutively expressed in multiple types of tumors, including haematological malignancies, suggesting that mechanism(s) of tumor escaping from NKG2D/NKG2DL-mediated immune surveillance may exist. Recently, it was reported that NKG2D ligand-expressing tumors evade immune control via proteolytic cleavage of the ligands from cancer cell surface in a soluble form [9], [10]. ADAM- and matrix metalloproteases cleaved soluble NKG2DL are believed to bind to the receptor, down-regulate its surface expression on circulating NK- and T cells and, thus, suppress the NKG2D-dependent pathway of cytotoxicity [9], [11]. Additionally, we and others have shown a novel mechanism for bioactive “soluble” NKG2DL secretion as membrane-bound molecules on the surface of normal- and/or tumor-cell exosomes [12]–[15].

Exosomes are specialized 30–100 nanometer-sized lipid-rich membrane-bound vesicles, actively formed and secreted through the endosomal compartment of a variety of living cells including a wide range of tumors [16]. Exosomes can be regarded as “messengers”, carrying surface- and luminal proteins to be exchanged between cells. The protein composition and functions of exosomes are determined by the cell types that produce them [16]. Exosomes also contain and are capable of intercellular transport of functional mRNA and microRNA that can epigenetically reprogram recipient cells [17]. Despite limited understanding of the exosome function *in vivo*, their capacity to modulate immunity is the feature with the greatest impact on cancer establishment and spreading. Cancer exosomes are enriched in tumor-associated antigens and can be used in diagnosis of malignancies [17], [18]. It has been shown *in vitro* that these exosomes can deliver tumor-associated antigens to the dendritic cells thus boosting anti-cancer immunity [19]. In contrast to the proposed immune activation stands the fact that cancer patients, in particular those with malignant effusions such as ascites, produce enormous amounts of exosomes *in vivo* and, instead of boosted anti-cancer immunity, they succumb to the cancer with a deranged immune system. Increasing clinical and experimental evidence shows that cancer cells produce exosomes which affect cytotoxic ability of NK- and T cells and thus assist cancers in their immune evasion. Consequently, tumor-derived exosomes might be vehicles for immunosuppression with negative impact on the immune system of cancer patients and their effects should be taken in consideration when designing treatment for cancer patients [20].

Despite the promising leukemia treatment programs of high-dose chemotherapy and stem cell transplantation relapses are frequent and often fatal. Accumulating evidence has shown that the immune escape of leukemia may be related to inadequate NK cell function such as low NK cell numbers and impaired cytotoxicity. The relevance of the NKG2D/NKG2DL system for the immune surveillance in patients with leukemia/lymphoma was previously described. It was shown that tumor cells extracted from different types of leukemia/lymphoma expressed heterogeneous levels of NKG2DL which rendered them susceptible to NK cell-mediated lysis in an NKG2D-dependent manner [8], [21].

Here, we investigated the exosome-mediated release of NKG2DL under steady-state and stress (specifically thermal and oxidative stress) conditions using the leukemia/lymphoma T- and B-cell lines Jurkat and Raji as hematopoietic malignancy models. We report that cellular stress significantly enhances the secretion of NKG2D-ligand bearing exosomes by tumor cells providing a higher amount of membrane-bound “soluble” form of NKG2DL. Our functional studies demonstrate that NKG2DL-carrying exosomes abrogate NKG2D-mediated NK-cell cytotoxicity and, thus, might contribute to the immune evasion of leukemia/lymphoma cells. Our results imply a novel exosome-based mechanism that might be another explanation for the observed NKG2D-dependent impairment of NK-cell function in patients with hematologic malignancies.

## Materials and Methods

### Cell cultures and stress induction

Human T cell leukemia Jurkat- and B cell leukemia/lymphoma Raji cell lines, purchased from ATCC, were cultured in RPMI 1640 (Gibco, Invitrogen) supplemented with penicillin/streptomycin, 10% heat-inactivated FCS and 2 mM L-glutamine at 37°C, 5% CO_2_ and 95% humidity designated as culture at a steady-state condition. Cultured cells were subjected to heat stress at 40°C for 1 h in water bath, followed by 2 h recovery at 37°C, 5% CO_2_ in humidity. For oxidative stress, cells were treated for 2 h with 100 µM and 50 µM H_2_O_2_ for Jurkat and Raji cells, respectively, at normal culture conditions. For exosome production, cells were seeded at10^6^ cells/ml, cultured 24 h before stress in complete medium with ultracentrifuged FCS, and allowed to recover for 2 h before a supernatant collection for exosome isolation was performed. During all experiments, cell viability was ≥90%. Enhancement of HSP70 mRNA was used as a positive control for the experimental stress conditions.

### Antibodies

The antibodies used in this study were as follows: anti-MIC mAb clone 6D4 and anti-NKG2D mAb clone 1D11 from BD Biosciences; isotype-matched control mAbs IgG1, IgG2a, FITC-conjugated IgG1, PE-conjugated IgG2a, normal rabbit Ig, FITC-conjugated swine anti-rabbit IgG, PE-conjugated goat anti-mouse IgG from Dako Cytomation; anti-CD63 mAb (clone CLB-gran/12,435) from Fitzgerald Industries Intl; FITC-conjugated anti-CD63 mAb from Immunotec; peroxidase-conjugated Ab: rabbit anti-mouse IgG, goat anti-rabbit IgG, rabbit anti-goat from Jackson ImmunoResearch Laboratories; mAbs against MICA/B (clone159207), MICA (clone159227), MICB (clone 236511), ULBP3 (clone 166510), PE-conjugated anti-ULBP1 (170818), PE-conjugated anti-ULBP2 (165903) and PE-conjugated goat-anti mouse IgG from R&D Systems; mAbs against CD63 (clone MX-49.129.5), HSP70 (clone W27), rabbit anti-human Abs against ULBP1 (clone H-46), ULBP2 (clone H-48), ULBP3 (clone H-45), goat anti-human MICA/B (clones E16 and G20) from Santa Cruz Biotechnology.

### Total RNA extraction and real-time quantitative RT- PCR

RNA was extracted from 3×10^6^ cells by Acid Guanidium Thiocyanate-Phenol-Chloroform extraction method as previously used [14], [15]. Reverse transcription was performed with random hexamers (Applied Biosystems), MULV reverse transcriptase (Promega), dNTPs (Promega) and 1 U RNAse inhibitors (Promega), at 42°C for 15 min, followed by denaturation at 99°C for 5 min. ULBPs and MICA/B were amplified on ABI PRISM 7700 by TaqMan Gene Expression Plate (I) protocol (PE Applied Biosystems). Primers and probe sequences were as previously described [15]. 18S rRNA was used as an endogenous control. Cycling conditions were as follows: 50°C for 2 min and 95°C for 10 min, followed by 40 cycles of 95°C for 5 s and 60°C for 1 min. The amplified mRNA was presented as relative quantities measured by n-fold increase of the amplification signal in stressed culture condition compared to the one in steady-state culture conditions ( = 1). Amplification of mRNA to HSP70 was used as a positive control to estimate the efficiency of the experimental stress conditions.

### Isolation of exosomes from cell culture supernatants

Supernatants were collected from cell culture after 24 h. Cells were spun down at 300× g and the supernatant was used for exosome preparation. Cell debris was removed by centrifugation at 4000× g for 30 min and 10000× g for 35 min. The supernatant was filtered through a 0.2 µm filter and ultracentifuged at 110,000× g for 2 h and the pellet was collected and resuspended. Exosomes were purified by ultracentrifugation on 20% and 40% discontinous sucrose gradient and subsequently washed with sterile filtered PBS. The samples were resuspended in PBS or RIPA buffer supplemented with protease inhibitor cocktail (Complete Mini: Roche Diagnostic). The exosome yield was measured with Micro BCA Protein Assay Kit (Pierce) [22] and Vybrand DiI staining (Molecular Probes) as previously described [23] and kept in −80°C until further use.

### Immunofluorescent staining and flow cytometry of cell lines

For cell surface staining, 500,000 cells were suspended in PBS containing 0.2% BSA and 0.02% NaN_3_, and incubated with appropriate concentrations of primary mAbs for 45 min on ice with constant shaking. After the incubation, the cells were centrifuged through a layer of 50 µl FCS, followed by two more washes. The cells were then incubated with secondary antibodies of FITC- or PE-labeled F(ab′)_2_ fragments of goat anti-mouse IgG for 45 min in darkness followed by washing steps. For double staining, directly conjugated antibodies were used in the last incubation step. Isotype-matched irrelevant mAbs were used as negative controls. For surface and intracellular staining, prior to the staining procedure the cells were fixed and permeabilized in 2% paraformaldehyde supplemented with 0.5% saponin for 20 min at r t, followed by incubation with 50 mM glycine and 10% human serum to block free aldehyde groups. After staining the cells were analysed on FACScan (BD Biosciences) using CellQuest software.

### Immunofluorescent staining and flow cytometry of NKG2D ligand expression on the surface of latex bead-coupled exosomes

Surfactant-free ultra-clean 4-µm sulphate latex microbeads (Interfacial Dynamics) were coated with mAbs against NKG2D ligands or CD63 rotating over night at 4°C according to the manufacturer's instructions. After washing and blocking of uncoupled sites with glycine and BSA, purified exosomes from equal volume of supernatant from the same number of cultured stressed and unstressed cells were added and incubated overnight with end-to-end rotation. The NKG2D ligand expression of bead-bound exosomes was revealed by immunofluorescent staining as described above using FITC-coupled anti-CD63 (Immunotech) or PE-coupled ULBP1, ULBP2 and ULBP3 mAbs (R&D Systems). Isotype-matched irrelevant mAbs were used in negative controls. A minimum of 10^4^ beads per sample were analysed on FACScan (BD Biosciences) using CellQuest software.

### Western blot

Exosomes isolated from cell culture supernatants were solubilised in RIPA buffer (Pierce), separated by SDS-PAGE on 12% polyacrylamide gels and transferred onto a polyvinylidene diflouride membrane (PVDF) (GE Healthcare). The membranes were blocked in 3–5% blocking reagent (GE Healthcare) in PBS-Tween (PBST) for 1 h at r t and incubated with respective Abs for CD63 and NKG2D ligands in 0.5–1% blocking reagent in PBST over night at 4°C. After 3×5 min washing in PBST the peroxidase-conjugated secondary Ab was applied at 1∶40,000 dilution in 1–2% blocking agent in PBST for 1 h at r t. After 3×5 min PBST- and 3×5 min H_2_O washes, the bands were detected by Amersham ECL plus and developed on Amersham ECL developing film (GE Healthcare). Protein bands of CD63 and NKG2D ligands from exosomes secreted by stressed and steady-state cultured cells were quantified by densitometric analysis (Image Quant 5.1) of autographs created from the Western blot assays.

### Electron microscopy of isolated exosomes

Negative contrast staining and immunoelectron microscopy (IEM) were used for analyses of the exosome morphology and surface expression of NKG2DL. The procedure of staining was performed as described elsewhere [15]. In brief, after adsorption to formvar/carbon-coated nickel grids the exosomes were fixed with 2% paraformaldehyde and either stained by negative contrast with 1.9% methyl cellulose containing 0.3% uranyl acetate or incubated with various monoclonal or polyclonal antibodies and isotype- matched controls for 1 h in wet chamber for IEM. After washing 5 or 10 nm gold particle-conjugated secondary antibodies were applied for 1 h. Finally, the samples were negatively stained as described and analysed in a Zeiss EM 900 electron microscope.

### Cytotoxicity assay

NK-cell-mediated cytotoxicity was measured by CytoTox 96 Non-Radioactive Cytotoxicity Assay (Promega) according to the manufacturer's instructions. The assay measures release of cytoplasmic lactate dehydrogenase in the culture medium as a result of cell lysis. The NKG2D-ligand expressing K562 cells [15] were used as targets and PBMC isolated from healthy donors were used as effector cells in an effector-target ratio of 40∶1. The effector and target cells were incubated for 4 h at 37°C alone or in the presence of Jurkat or Raji exosomes, Ab-blocked exosomes, and Ab-blocked target- or effector cells as previously described [15]. In all experiments, the exosomes were isolated from supernatant produced by the same number of cultured stressed or unstressed cells, for Jurkat 40.10^6^ cells and for Raji 24.10^6^. For blocking of the NKG2D receptor on the effector cells or the NKG2D ligands on the exosomes NKG2D mAb (clone 1D11, BD Bioscience), CD63 mAb (clone MX-49.129.5, Santa Cruz) or a cocktail of NKG2DL Abs; MICA/B, clone E16; ULBP1, clone H-46; ULBP2, clone H-48, all from Santa Cruz) were used. Blocking of the exosomes with single anti-CD63 mAb or with a cocktail of Abs against NKG2D ligands gave similar results. The anti-CD63 mAb was used as comparison to exclude that the observed blocking by the Ab-cocktail was not due to steric hindrance. The specific lysis was calculated by a standard formula according to the manufacturer's instructions.

### Statistical analysis

The statistical significance, calculated by Student's t test is presented in the figures. A value of p<0.05 was considered significant.

## Results

### The effect of thermal and oxidative stress on NKG2DL mRNA and protein expression in Jurkat and Raji cells shows cell-line specific differences and enhancement of intracellular protein expression

**Figure 1:**
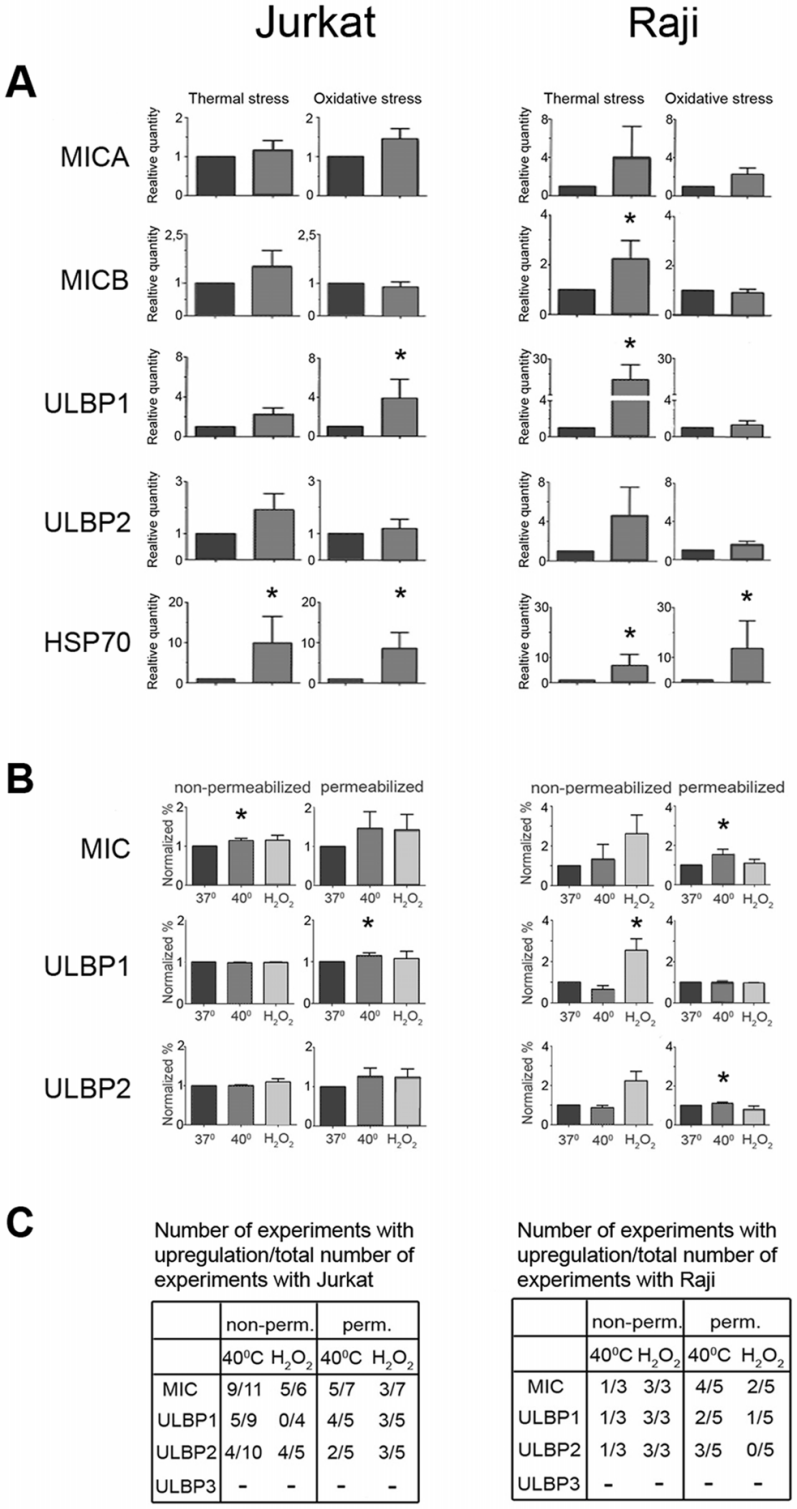
Effect of stress on NKG2DL expression in Jurkat and Raji shows cell line-specific differences. **A.** NKG2DL mRNA expression before and after thermal- and oxidative stress measured by real-time quantitative RT-PCR. The relative mRNA expression under stress conditions was normalized to the mRNA expression in steady-state culture ( = 1, dark staples). The efficacy of stress treatment was assessed by measurement of mRNA for HSP70. 18S rRNA was used as endogenous control. **B.** Immunoflow cytometry staining of untreated and stressed Jurkat and Raji cells with mAbs against MICA/B and ULBP1-2. Isotype matched mAbs were used as negative controls and the expression was normalized to the expression in untreated cells. **C.** Tables summarizing the number of immunoflow cytometry experiments with stress-induced up-regulation of NKG2D ligands. * = statistical significance, p<0.05.

Messenger RNA and protein expression of MICA/B and ULBP 1–3 in Jurkat and Raji cells following stress was assessed by real-time quantitative RT-PCR and immunoflow cytometry. The results of mRNA assessment are summarized in Figure 1A. Up- regulation of mRNA for HSP70 was used as a control of the experimental stress conditions. Both cell lines constitutively expressed mRNA for MICA, MICB, ULBP1 and ULBP2 and up regulated the message after cellular stress. We did not find ULBP3 mRNA expression at steady state or after thermal and oxidative stress. These results are in line with the report by Nückel et al. [24] that cancer cells from chronic B cell leukemia patients lacked ULBP3 mRNA. Lanca et al. [25] reported similar results for ULBP3 mRNA in Jurkat cells but a low ULBP3 mRNA expression in Raji. Some cell line-specific differences could be noted. In Jurkat cells the NKG2DL mRNA expression was approximately equally up-regulated by both types of stress. Raji cells were generally more susceptible to NKG2DL mRNA up-regulation compared to Jurkat and reached significantly higher levels of mRNA under thermal stress compared to oxidative stress.

Further, we investigated the NKG2DL protein expression by flow cytometry and the results, normalized to the expression in cells cultured at steady state conditions, are presented in Figure 1B and the number of experiments is summarized in Figure 1C. Both cell lines expressed MICA/B, ULBP1 and ULBP2 on the cell surface and intracellularly as shown by total protein staining of permeabilized cells. ULBP3 protein was absent, reflecting our PCR finding. In Jurkat cells, there was a significant up-regulation of surface MICA/B expression after thermal stress. The normalized total protein expression was generally higher after thermal stress reaching statistical significance for ULBP1 (Figure 1B). In Raji cells, the normalized surface protein expression was mainly enhanced by oxidative stress compared to thermal stress. Thermal stress did not affect the surface expression, however, the normalized total protein expression was significantly increased suggesting that NKG2DL might be mainly located inside the cells (Figure 1B).

In conclusion, our NKG2DL mRNA- and protein expression assessment showed that 1) both cell lines respond to thermal and oxidative stress by up-regulation of mRNA for some NKG2DL and show cell-line specific differences; 2) NKG2DL proteins are expressed both on the cell surface and intracellularly; 3) Raji cells seem to be more sensitive to thermal than oxidative stress as reflected by an up-regulation of mRNA transcripts and enhanced intracellular NKG2DL protein expression.

### Assessment of NKG2DL expression on the surface of exosomes secreted by Jurkat and Raji cells under steady state and stressed culture conditions by electron microscopy

**Figure 2:**
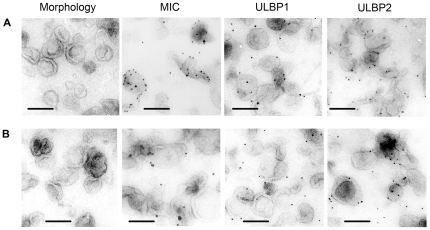
Electron microscopy analyses of secreted exosomes by Jurkat and Raji cells. Negative contrast staining showing typical exosomal morphology and electron micrographs illustrating immunogold staining of the NKG2D ligands MIC, ULBP1 and 2 of exosomes isolated from **A.** Jurkat and **B.** Raji. Bars represent 100 nm.

Isolated exosomes from steady state and stressed culture conditions were subjected to negative contrast staining to assess their morphology and purification grade, and thereafter to immunogold staining for NKG2DL and the exosomal marker CD63. Similar results were obtained for both cell lines (Figure 2). The negative contrast staining showed a pure population of microvesicles with typical cup-shaped exosomal morphology, varying in size between 40–100 nm, the majority around 90–100 nm. Besides morphology and size, the exosomal nature of the microvesicles was confirmed by CD63 immunogold staining (not shown). Thermal and oxidative stress can cause cell death, thus, precautions were taken to use cells in excellent conditions throughout all experiments and to exclude cell debris and apoptotic bodies from the exosomal preparation by the use of sucrose gradient in the isolation procedure. Electron microscopy demonstrated a pure exosomal population that was not affected in morphology and size by the stress conditions (not shown). Staining with anti-NKG2DL antibodies revealed that exosomes produced by Jurkat and Raji cells expressed MICA/B and ULBP1 and 2 on their surface. The results of the electron microscopy are illustrated with representative photomicrographs of exosomes from Jurkat (Figure 2A) and Raji (Figure 2B) cells under steady state conditions.

### Thermal and oxidative stress significantly increases the exosome secretion by Jurkat and Raji leukemia/lymphoma cells

**Figure 3:**
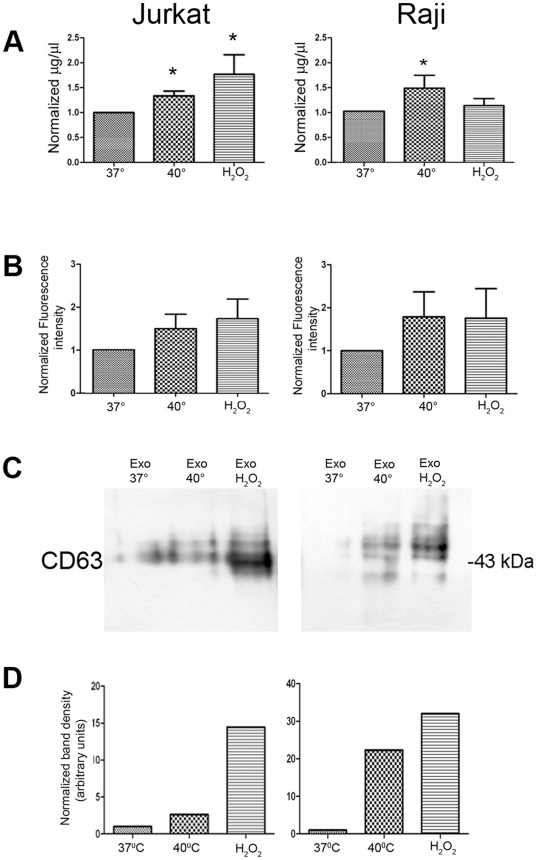
Thermal- and oxidative stress increases the release of exosomes by Jurkat and Raji cells. Exosomes were isolated with sequential centrifugations and sucrose gradient from supernatants from the same number of untreated and stressed cells. Measurement of isolated exosomes by **A.** BCA protein assay, **B.** fluorescence measurement of Vybrant DiI stainings of exosomal lipid membranes, **C.** western blot for the exosomal marker CD63. **D.** Densitometry for the exosomal marker CD63, the density of the bands was normalized to the bands from exosomes released by cells cultured at steady-state conditions ( = 1). * = statistical significance, p<0.05.

In the next step, we investigated whether thermal and oxidative stress also affected the quantity of exosomes secreted by Jurkat and Raji cells. Using sucrose gradient ultracentrifugation, we isolated exosomes from cell culture supernatants produced by equal amount of Jurkat and Raji cells cultured under steady state and stress conditions, and measured the exosomal yield by three different methods. At present, there is no well-established and recognized method for exosome quantification. The most frequently used methods are based on total exosomal protein measurement by BCA assay and densitometric analysis of Western blot bands [22]. Recently, fluorescence intensity measurement of exosomes labeled with lipophilic fluorescent dyes has also been suggested and used [23]. To enhance the reliability of our measurements we used all three methods - BCA protein assay, fluorescence intensity after exosomal membrane staining with Vybrand DiI and densitometry of Western blots. The results are summarized in Figure 3. Under stress, the exosome secretion from both cell lines was increased as measured by all three methods, reaching a statistical significance in the measurement by BCA assay (Figure 3A, n = 11). A clear tendency of increased exosome quantity was seen by fluorescence intensity (Figure 3B, n = 5). Figure 3C is a Western blot of one representative experiment for the exosomal marker CD63 reflecting the higher protein amount under stressed conditions. Figure 3D shows an increased band density of CD63 after thermal- and oxidative stress, reaching 3-fold increase by thermal- and 15-fold increase by oxidative stress for Jurkat exosomes, and 22-fold increase by thermal- and 32-fold increase by oxidative stress for Raji exosomes. These measurements suggest that oxidative stress seems to enhance exosome secretion to a higher degree than thermal stress, and that Raji cell line seems to be more susceptible to stress-mediated up-regulation of exosome secretion compared to Jurkat. We conclude that cellular stress can up-regulate exosome secretion by both T- and B-cell leukemia/lymphoma cells.

### Secretion of exosomal form of NKG2DL by Jurkat and Raji cells is increased under stress conditions

**Figure 4:**
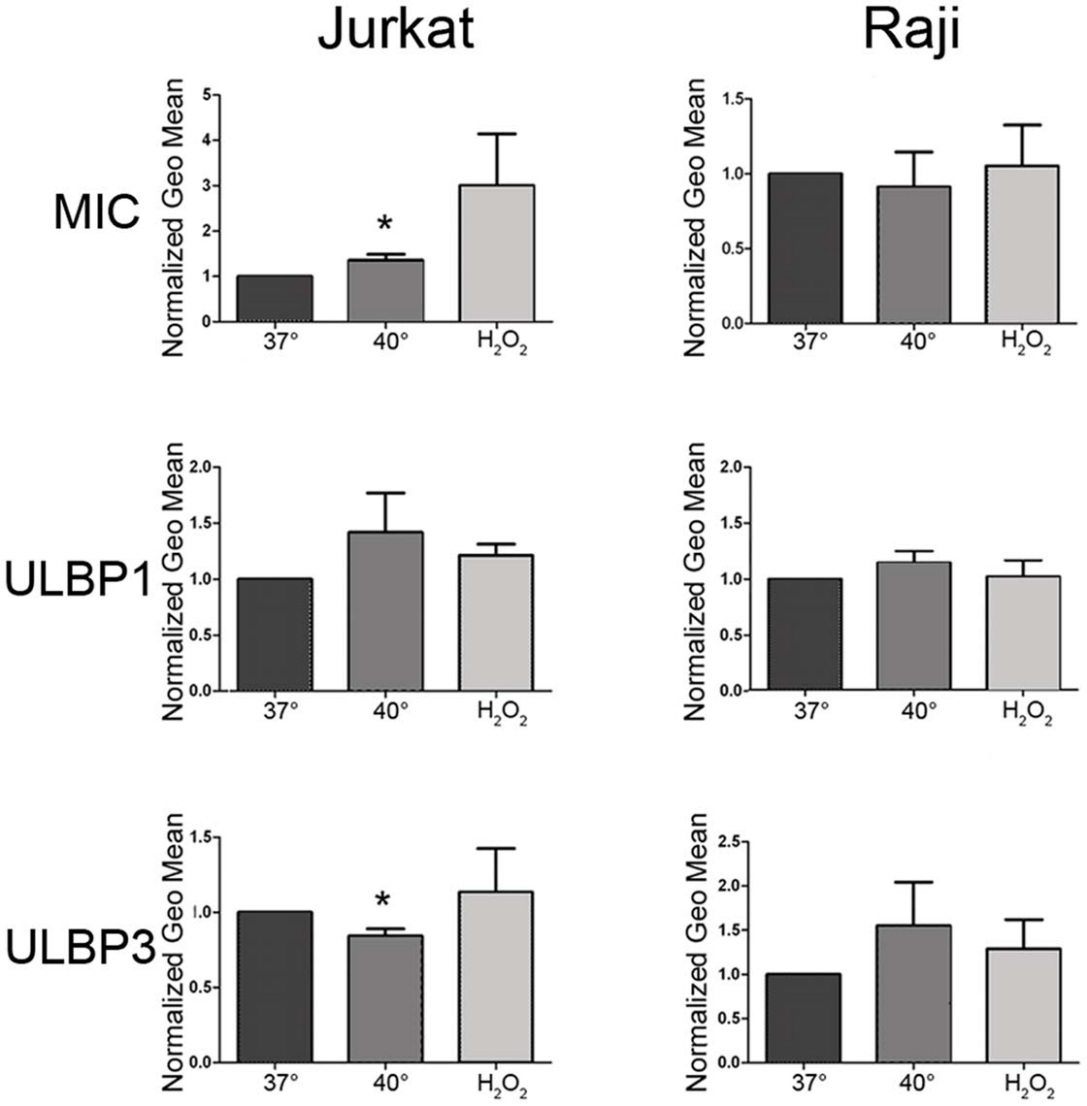
Stress-increased exosomal NKG2DL enhance the suppressive effect of Jurkat and Raji exosomes on NK-cell cytotoxicity. Immunoflow cytometry of latex microbead-captured exosomes released from unstressed and stressed cells stained for NKG2D ligands or the exosomal marker CD63. Geo mean of fluorescence intensity is normalized to steady state culture conditions at 37°C ( = 1). * = statistical significance, p<0.05.

Using three different measurements, we could estimate that stress increased the amount of secreted exosomes. By electron microscopy, we showed that these exosomes expressed NKG2DL on their surface. Summarizing these experiments, it is logical to anticipate that the total amount of exosomal NKG2D ligands under stress conditions should also be increased. To prove this suggestion, NKG2DL expression was assessed by immunofluorescence staining and flow cytometry of exosomes coupled to latex beads. Exosomes were isolated from supernatant of equal amount of cells cultured under steady state and stressed conditions, resuspended to equal volume in PBS and coupled to surfactant-free Abs-coated latex microbeads. The coupled exosomes were stained for NKG2DL. The obtained results showed cell line-specific differences and are summarized in Figure 4. In Jurkat cell-derived exosomes, MIC expression was significantly up regulated after thermal stress in 3/3 experiments, and the same tendency was found after oxidative stress. Exosomal expression of ULBP1 was up-regulated by thermal- and oxidative stress in 2 out of 3 and 3/3 experiments respectively, while ULBP2 expression was not affected by oxidative stress and down regulated after thermal stress (Figure 4). In Raji cell-derived exosomes, the highest up-regulation was observed with thermal stress for ULBP2 (n = 3/3experiments) followed by ULBP1 (n = 3/4 experiments), while MIC expression was only slightly affected by thermal or oxidative stress. In common, it seemed that thermal and oxidative stress can increase the total amount of exosome-expressed NKG2DL proteins.

### Thermal and oxidative stress enhances the suppressive effect of NKG2D ligand-bearing exosomes on NK-cell mediated cytotoxic response

**Figure 5:**
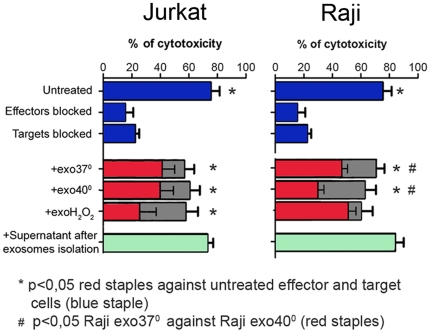
Stress enhances the immunosuppressive effect of NKG2DL-bearing exosomes. NK-cell cytotoxicity assay using PBMC from healthy donors and K562 targets at an E∶T ratio 40∶1. The cytotoxic effect was measured in the presence or absence of exosomes released from cells cultured under steady state or stressed conditions. The cytotoxic response of untreated or antibody-blocked effector and target cells are shown in blue staples. The suppression of cytotoxicity by native exosomes released from cells cultured in various conditions is shown in red staples. Gray staples underlayed under the red staples show reversal of cytotoxicity to normal levels when the exosomes were blocked with a cocktail of Abs against NKG2DL or with Abs against the exosomal marker CD63. Green staple shows the level of cytotoxicity in the presence of used supernatant after exosome isolation indicating that the suppressive effect was associated with the exosomal fraction. * and ^#^ indicates statistical significance, p<0.05.

It has previously been reported by other and us that NKG2DL-bearing exosomes can impair the cytotoxic function of NK cells [12]–[15]. Therefore, as a next step, we investigated if the increased secretion of NKG2DL-bearing exosomes had consequences for the cognate receptor-mediated killing *in vitro*. The experiments were done with the NKG2D ligand expressing target cells K562 in effector∶target ratio of 40∶1 and in the presence or absence of exosomes, which were isolated from equal number of cultured cell under thermal or oxidative stress conditions. PBMC from healthy donors, containing NKG2D-receptor expressing NK-, CD8^+^- and γδT cells were used as effector cells. Cytotoxicity was assessed in untreated effector cells or effector cells pretreated with native exosomes, Ab-blocked exosomes, Ab-blocked target- or Ab-blocked effector cells and supernatant after exosome isolation, as described in Material and Methods. The results are summarized in Figure 5. As can be seen, there was a significant downregulation of the cytotoxic response with reduction by approximately 50% in the presence of native exosomes isolated from Jurkat and Raji cells cultured under steady state conditions (Figure 5, red staples). Moreover, the suppression was enhanced when the exosomes were from cells cultured in stressed conditions. An interesting observation is that in Jurkat cells, enhanced suppression was observed in exosomes from oxidative stress conditions, which was the stress that caused the highest significant increase of exosome secretion as illustrated in Figure 3. In contrast, thermal stress caused significant increase of exosome secretion in Raji cells (Figure 3). Accordingly, we found the highest suppression of cytotoxicity when Raji exosomes produced under thermal stress conditions were used (Figure 5, red staples). The suppression of cytotoxicity was reversed when the exosomes were pretreated with blocking Abs as illustrated in the gray staples behind the red ones (Figure 5). No effect was observed when used supernatant after exosome isolation was tested, indicating that the specific suppression of cytotoxicity was found in the exosomal fraction (Figure 5, green staples).

In conclusion, our cytotoxicity experiments suggest that the suppressive effect of the exosomes on the NK-cell cytotoxicity showed cell line-specific differences and was enhanced by the stress culture conditions that triggered increased exosome amount.

## Discussion

In this report we have used Jurkat and Raji cell lines as a model for studies of exosome-mediated NKG2DL secretion by T – and B cell leukemia/lymphoma cells under stress conditions for the following reasons: i) these rapidly progressing blood malignancies have poor prognosis due to broken immune surveillance caused by inadequate NK-cell function; ii) exosome secretion is a constitutive feature of many human malignancies; iii) tumor-derived exosomes are known to express NKG2DL and interfere with the powerful cytotoxic NKG2D receptor-ligand pathway that is instrumental for NK-cell function; iv) the treatment regimens of these malignancies include thermotherapy and heavy cytostatic treatment both of which expose the body to massive cellular stress; v) a comprehensive clinical study by Nückel et al. [24] showed that soluble NKG2DL were present in the peripheral blood of patients with chronic B-cell leukemia and related to a prognostic significance.

Our results showed that: i) leukemia/lymphoma cells constitutively expressed mRNA and proteins for the NKG2D ligands MICA/B, ULBP1 and ULBP2 and up-regulate their expression under thermal and oxidative stress; ii) leukemia/lymphoma cells constitutively secreted exosomes and the exosome secretion was significantly increased by thermal and oxidative stress; iii) the leukemia/lymphoma cell-derived exosomes carried NKG2DL of both the MIC and ULBP families; iv) the increased amount of NKG2DL-bearing exosomes enhanced the suppression of the NKG2D-dependent NK cell cytotoxicity, promoting an immune escape for these cells.

Despite the accumulated reports about the nature of stress signals inducing NKG2DL expression, only limited information about the precise mechanisms that lead to ligands' up-regulation in cancer is available. The promoter elements for transcriptional regulation of the expression of these ligands are not yet fully apprehended. MICA/B molecule expression is regulated by promoter elements similar to those of heat shock protein 70 gene while the transcriptional regulation of other NKG2D ligands remains obscure [1], [3], [8]. We chose the up-regulation of HSP70 transcripts as a positive control to estimate the effectiveness of stress induction in our experimental procedures. Previously, it has been reported that heat shock–induced transcriptional activation has not been observed for ULBPs [1], [3]. However, in this study we demonstrated heat shock-induced mRNA up-regulation and protein expression for both MIC and ULBP1 and 2 in a cell line-specific manner. Furthermore, these NKG2D ligands were expressed on exosomes secreted by cells cultured in steady state or under stressed conditions. At present, we cannot explain the reason for this discrepancy, maybe it has to do with differences in the antibody specificities, the cell lines and/or the experimental conditions. We did not find mRNA transcription and protein expression for ULBP3 which is in line with other reports [24], [25].

It is a well established fact that cancer patients carry tumor-secreted exosomes in peripheral blood and other bodily fluids as well as in various malignant effusions [18], [20]. The role of exosomes in cancer patients has been a controversial issue. From one side, convincing *in vitro* data have suggested that tumor derived exosomes could function as carriers of tumor antigens that were efficiently delivered to dendritic cells for antigen presentation, resulting in activation of anti-tumor immune response [19], [26], [27]. From another side, equally convincing reports have shown that tumor-derived exosomes may exert suppressive effect on the immune system interfering with various immune responses such as lymphocyte proliferation, T-cell receptor signalling and NK-cell cytotoxicity [12], [13], [28], [29]. Clayton et al. [12], [13] showed that exosomes released by breast-, mesotelioma and prostate cancer cell lines expressed NKG2D ligands with ability to down modulate the cognate NK cell receptor and impair the cytotoxic anti-cancer immune response. Moreover, in our studies of human normal pregnancy, we found that placenta secreted NKG2DL-expressing exosomes with similar suppressive effect on NK cytotoxicity providing immune escape of the fetus. [14], [15], [30].

At present, two forms of soluble NKG2D ligands have been described, a truncated ectodomain-limited soluble form produced by proteinase-induced cleavage of cell-surface expressed NKG2DL [11], [31], [32] and NKG2D ligand-bearing exosomes [12]–[15]. Cleavage of NKG2DL from the cell membrane is one way to reduce their expression on the plasma membrane leading to reduced susceptibility to NKG2D receptor-mediated cytotoxicity. In parallel, intracellular retention of the NKG2DL and sorting them to multivesicular bodies in the endosomal compartment for exosome release is another way to escape NKG2D receptor recognition [15]. Presence of biologically-active molecules in two soluble forms, a proteinase-cleaved and exosomal membrane-bound form, is not a new phenomenon. A similar situation exists for FasL that can be found in two forms with different biological properties - a soluble form produced by proteinase-cleavage of its membranal form, and on secreted exosomes [33], [34]. Both forms of soluble NKG2DL exist side by side in tumor settings and have been reported to cause NKG2D receptor down-regulation [13], [31], [32], [35]. The exosomal form provides multivalent expression of NKG2DL with preserved membrane-bound molecular structure and has been shown *in vitro* to be a more potent way for suppression of cytotoxicity compared to proteinase cleaved and thus truncated ligands [13], [35]. Our study demonstrates for the first time that thermal- and oxidative stress enhance the exosome-mediated secretion of NKG2D ligands. As a consequence, the suppression of NKG2D-mediated cytotoxicity was aggravated, which might promote immune escape of the leukemia/lymphoma cells.

Oxidative stress and hyperthermia are usually used as an adjunctive therapy alongside conventional cancer treatments. It was recently reported that hyperthermia can suppress the lytic potential of NK cells via down-regulation of perforin/granzyme B expression [36]. Our results suggest that, in addition to the suppressed cytolytic machinery of the effector cells, thermal stress might further augment the dysfunction of the NK cells by down-regulating their killing ability via increased secretion of immunosuppressive, NKG2DL-carrying tumor exosomes. Thus, we suggest that efforts should be focused not only on the soluble NKG2DL cleaved from the cell surface of cancer cells but also on the exosomal form of these ligands to include the exosome-driven immune suppression as well.

In conclusion, the present report confirms and reinforces the importance of the NKG2DL-expressing tumor exosomes as inhibitory vehicles mediating tumor escape from cytotoxic immune attack. Furthermore, we found that stress enhances tumor exosome secretion in general and causes an increase of exosome-carried NKG2D ligands in particular, resulting in suppression of NKG2D-mediated cytotoxicity. These results might partly provide a mechanistical explanation of the clinically observed NK-cell dysfunction in patients suffering from leukemia/lymphoma which could be further impaired in conditions of cellular stress. Our results should be taken into account when designing cytostatic and hyperthermal anti-cancer therapy.
